# Comparing the clinical and cost-effectiveness of remote (telehealth and online) cognitive behavioral therapy-based treatments for high-impact chronic pain relative to usual care: study protocol for the RESOLVE multisite randomized control trial

**DOI:** 10.1186/s13063-023-07165-8

**Published:** 2023-03-16

**Authors:** Meghan Mayhew, Benjamin H. Balderson, Andrea J. Cook, John F. Dickerson, Charles R. Elder, Alison J. Firemark, Irina V. Haller, Morgan Justice, Francis J. Keefe, Carmit K. McMullen, Maureen C. O’Keeffe-Rosetti, Ashli A. Owen-Smith, Christine Rini, Jennifer L. Schneider, Michael Von Korff, Laura D. Wandner, Lynn L. DeBar

**Affiliations:** 1 Kaiser Permanente Center for Health Research, Portland, USA; 2 Kaiser Permanente Washington Health Research Institute, Seattle, USA; 3 Essentia Institute of Rural Health, Duluth, USA; 4 Department of Psychiatry and Behavioral Sciences, Duke University Medical Center, Durham, USA; 5 Georgia State University and Center for Health Research and Evaluation Kaiser Permanente Georgia, Atlanta, USA; 6 Department of Medical Social Sciences, Northwestern University Feinberg School of Medicine and Robert H. Lurie Comprehensive Cancer Center of Northwestern University, Chicago, USA; 7 National Institute of Neurological Disorders and Stroke, Bethesda, USA

**Keywords:** Chronic pain, Nonpharmacologic treatment, Pragmatic trial, Cognitive behavioral therapy, Telehealth

## Abstract

**Background:**

Cognitive behavioral therapy for chronic pain (CBT-CP) is an effective but underused treatment for high-impact chronic pain. Increased access to CBT-CP services for pain is of critical public health importance, particularly for rural and medically underserved populations who have limited access due to these services being concentrated in urban and high income areas. Making CBT-CP widely available and more affordable could reduce barriers to CBT-CP use.

**Methods:**

As part of the National Institutes of Health Helping to End Addiction Long-term® (NIH HEAL) initiative, we designed and implemented a comparative effectiveness, 3-arm randomized control trial comparing remotely delivered telephonic/video and online CBT-CP-based services to usual care for patients with high-impact chronic pain. The RESOLVE trial is being conducted in 4 large integrated healthcare systems located in Minnesota, Georgia, Oregon, and Washington state and includes demographically diverse populations residing in urban and rural areas. The trial compares (1) an 8-session, one-on-one, professionally delivered telephonic/video CBT-CP program; and (2) a previously developed and tested 8-session online CBT-CP-based program (painTRAINER) to (3) usual care augmented by a written guide for chronic pain management. Participants are followed for 1 year post-allocation and are assessed at baseline, and 3, 6, and 12 months post-allocation. The primary outcome is minimal clinically important difference (MCID; ≥ 30% reduction) in pain severity (composite of pain intensity and pain-related interference) assessed by a modified 11-item version of the Brief Pain Inventory-Short Form at 3 months. Secondary outcomes include pain severity, pain intensity, and pain-related interference scores, quality of life measures, and patient global impression of change at 3, 6, and 12 months. Cost-effectiveness is assessed by incremental cost per additional patient with MCID in primary outcome and by cost per quality-adjusted life year achieved. Outcome assessment is blinded to group assignment.

**Discussion:**

This large-scale trial provides a unique opportunity to rigorously evaluate and compare the clinical and cost-effectiveness of 2 relatively low-cost and scalable modalities for providing CBT-CP-based treatments to persons with high-impact chronic pain, including those residing in rural and other medically underserved areas with limited access to these services.

**Trial registration:**

ClinicalTrials.gov NCT04523714. This trial was registered on 24 August 2020.

## Administrative information

Note: the numbers in curly brackets in this protocol refer to SPIRIT checklist item numbers. The order of the items has been modified to group similar items (see http://www.equator-network.org/reporting-guidelines/spirit-2013-statement-defining-standard-protocol-items-for-clinical-trials/).

**Table:** 

**Title {1}**	Comparing the clinical and cost effectiveness of remote (telehealth and online) cognitive behavioral therapy-based treatments for high impact chronic pain relative to usual care: study protocol for the RESOLVE multisite randomized control trial
Trial registration {2a and 2b}	This trial was registered in ClinicalTrials.gov on 24 August 2020. Identifier number: NCT04523714 https://clinicaltrials.gov/ct2/show/NCT04523714
Protocol version {3}	10 November 2022; Version 5.0
Funding {4}	This research is supported by the National Institutes of Health (NIH), National Institute on Aging (NIA) through the NIH HEAL Initiative [UH3AG067493, PI: DeBar]. Research reported in this publication was also supported by the National Center for Advancing Translational Sciences, Trial Innovation Network under award number U24TR001597 (Clinical Coordinating Center), U24TR001608 (Data Coordinating Center), U24TR001609 (Recruitment Innovation Center), and U24TR001579 (Safety and Statistical Coordinating Center).
Author details {5a}	**Meghan Mayhew**, MPH, Kaiser Permanente Center for Health Research* (meghan.h.mayhew@kpchr.org) **Benjamin H. Balderson**, PhD, Kaiser Permanente Washington Health Research Institute **Andrea J. Cook**, PhD, Kaiser Permanente Washington Health Research Institute **John F. Dickerson**, PhD, Kaiser Permanente Center for Health Research **Charles R. Elder**, MD, MPH, Kaiser Permanente Center for Health Research **Alison J. Firemark**, MS, Kaiser Permanente Center for Health Research **Irina V. Haller**, PhD, MS, Essentia Institute of Rural Health **Morgan Justice**, MA, Kaiser Permanente Washington Health Research Institute **Francis J. Keefe**, PhD, Department of Psychiatry and Behavioral Sciences, Duke University Medical Center **Carmit K. McMullen**, PhD, Kaiser Permanente Center for Health Research **Maureen C. O’Keeffe-Rosetti**, MS, Kaiser Permanente Center for Health Research **Ashli A. Owen-Smith,** PhD, SM, Georgia State University and Center for Health Research and Evaluation Kaiser Permanente Georgia **Christine Rini**, PhD, Department of Medical Social Sciences, Northwestern University Feinberg School of Medicine and Robert H. Lurie Comprehensive Cancer Center of Northwestern University **Jennifer L. Schneider**, MPH, Kaiser Permanente Center for Health Research **Michael Von Korff**, ScD, Kaiser Permanente Washington Health Research Institute **Laura D. Wandner**, PhD, National Institute of Neurological Disorders and Stroke **Lynn L. DeBar**, PhD, MPH, Kaiser Permanente Center for Health Research ^*^Corresponding author
Name and contact information for the trial sponsor {5b}	National Institute on Aging (NIA), P.O. Box 8057, Gaithersburg, MD 20,898
Role of sponsor {5c}	The content is solely the responsibility of the authors and does not necessarily represent the official views of the NIH or its NIH HEAL Initiative.

## Introduction

### Background and rationale {6a}

Chronic pain is one of the most common, disabling, and costly public health problems in the USA [1] and a primary reason that patients seek medical care. About 1 in 10 US adults experience high-impact chronic pain, defined as pain that has lasted 3 months or longer and is accompanied by at least 1 major activity restriction, such as being unable to work outside the home, go to school, or do household chores [1–3]. Rural residency is associated with higher prevalence of high-impact chronic pain with complicating features such as depression and medical comorbidities [4, 5]. Rural residents with chronic pain report higher pain frequency and intensity and more pain-related disability and depression than urban residents with pain [6, 7]. Further, healthcare disparities between rural and urban areas are widely recognized with difficulties in availability, accessibility, and affordability of health services often disproportionately impacting rural residents [8]. These disparities are associated with higher risk of adverse outcomes and suboptimal pain management among rural patients with chronic pain.

Until relatively recently, opioids were the main, and often only, treatment provided for long-term chronic pain management despite limited evidence of effectiveness [9]. Opioid treatment-related addiction, overdose, and other harms resulting from widespread opioid use left an aftermath of adverse effects for patients, families, and communities [10, 11]. These consequences have been especially pronounced in rural communities where rates of non-medical prescription opioid use and overdose have been disproportionately higher than in urban areas [12, 13]. Consequently, primary care clinicians and their patients, especially in rural and medically underserved areas, urgently need increased access to viable non-opioid options and treatments for long-term chronic pain management.

Cognitive behavioral therapy (CBT) is the most widely accepted and effective non-pharmacotherapy treatment for chronic pain [1, 14, 15] and has been shown to benefit adults with low literacy in rural areas [16]. Cognitive behavioral therapy for chronic pain (CBT-CP) is based on the theory that patients’ beliefs, attitudes, behavior, and coping styles play central roles in mediating the impact of persistent pain on patients’ lives [17]. It focuses on helping patients develop and master skills to manage pain and the way pain affects their thoughts, feelings, and both physical and social activities in order to improve functioning and quality of life. Because CBT-CP focuses on learning, applying, and mastering coping skills, its effects potentially can be maintained long after treatment ends, without the negative side effects of opioids and other pain medications. The National Pain Strategy calls for wider implementation of CBT-CP self-management training [18], noting that despite evidence supporting its efficacy, CBT-CP implementation lags due to significant barriers including a paucity of trained providers and their concentration in higher socioeconomic status urban areas.

Outside Veterans Affairs (VA) healthcare systems [19], few clinicians are trained to deliver CBT-CP services in community practice. Despite the growing emphasis on standardized training and implementation methods, CBT-CP services often vary in content and quality [20]. In community settings, fidelity to best CBT-CP treatment practices is rarely assessed [21]. Frontline behavioral health providers offering CBT-CP generally have large caseloads and typically prioritize treatment for mental health disorders. These problems are especially critical in rural and medically underserved areas [6, 8, 22] where behavioral health and psychotherapy providers are scarce and few professionals are trained to deliver CBT-CP [23]. In addition, conventional CBT-CP programs can be difficult to access for patients with competing demands, limited time, and transportation or mobility barriers. Most CBT-CP programs require weekly in-person sessions of 50 min or more over 2 to 3 months, often in group formats with inflexible scheduling. Because of these and other barriers, patients referred for CBT-CP may not attend a sufficient number of sessions to receive an adequate treatment dose [24]. Further, the stigma associated with what are perceived to be “psychological” interventions can limit patient uptake [25, 26]. Providing CBT-CP by telephone, video visit, or an online program may help reduce access barriers, destigmatize its use, and encourage patients to view it as complementing conventional medical management of chronic pain. Lastly, primary care providers often lack familiarity with CBT-CP and may not have enough time to discuss CBT-CP with patients, lack ways to provide a compelling rationale for CBT-CP, or have difficulty explaining why a psychologically informed treatment may help manage a physical condition [27]. Moreover, few guidelines exist for determining which patients might benefit from CBT-CP [28–30] and in which circumstances the various formats for delivery of CBT-CP-based interventions (e.g., led by trained interventionist vs. self-guided online format) may be preferable [31].

Technologies to deliver and assist treatment such as telephone-based and online treatment programs [32–34] offer ways to increase access to evidence-based pain care, including CBT-CP and related self-management approaches [18, 35, 36]. Prior studies have demonstrated clinically meaningful benefits of telephone-based CBT-CP for pain-related outcomes in the context of multicomponent interventions [34, 37, 38]. Online CBT-CP treatment programs, which deliver training in a self-completed, interactive, web-based format and do not rely on or only sometimes include the involvement of a trained therapist, have demonstrated efficacy for improving pain and pain-related impairment as supported by multiple meta-analyses [36, 39, 40]. Although overall effect sizes are modest, these reviews suggest the potential impact of these treatment approaches with respect to lower costs and greater safety than pharmacologic pain treatments [36, 39, 40].

Additionally, remote services can reach large numbers of patients at lower costs per patient treated while overcoming system, patient, and clinician barriers [41]. Growing evidence suggests that remotely provided interventions can improve self-management and chronic disease management outcomes [42]. Due in part to the COVID-19 pandemic, telehealth services have rapidly expanded with a particular push towards video visits which best approximate traditional in-person visits, while provision of self-administered online services has also increased markedly due to lower costs and greater accessibility [43–45].

### Objectives {7}

The primary objective of the RESOLVE study is to determine the comparative effectiveness of 2, remotely delivered, CBT-based, interventions for chronic pain: (1) an 8-session health coach delivered telephonic/video conference one-on-one program and (2) an 8-session online self-completed program. Effectiveness will be assessed by comparing clinically meaningful improvements in pain severity, a composite measure of pain intensity and pain-related interference, among patients with high-impact chronic pain at 3 months post-allocation, relative to usual care and each other.

Secondary objectives of the study include examining whether the interventions have an enduring impact on pain severity at 6 and 12 months post-allocation as well as the interventions’ impact at 3, 6, and 12 months on pain intensity, pain-related interference, social role functioning, physical role functioning, and patient global impression of change. The cost and incremental cost-effectiveness of the 2 interventions compared to usual care and each other will also be assessed.

The RESOLVE study also includes qualitative interviews with participants, healthcare system leadership, and study staff. These data will be used to better understand the acceptability of CBT-CP-based interventions for patients and health systems, lessons learned during study implementation, and changes in usual care during the study period. Exploratory objectives also include assessing the impact of the interventions on long-term opioid use, comorbid symptomology (depression, anxiety, and sleep disturbance), and chronic pain grade.

### Trial design {8}

**Fig. 1:**
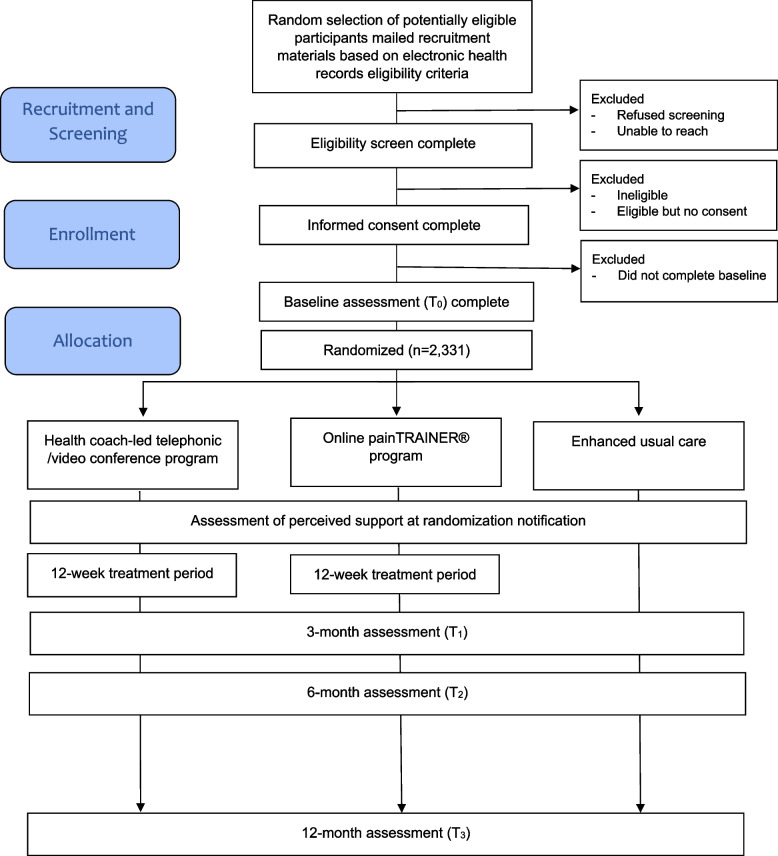
Participant flow

The RESOLVE study is a multisite, prospective, comparative effectiveness, phase III randomized controlled trial (RCT). The study uses a parallel group design in which study participants are randomized in equal ratio to 1 of 3 study groups. One group receives 8, one-on-one, CBT-CP sessions with a health coach via telephone or video conference to be completed within approximately 12 weeks from randomization. A second group receives an 8-session, online CBT-CP-based program that delivers pain coping skills training (painTRAINER®) to be completed within approximately 12 weeks from randomization. The content of the health coach CBT-CP sessions and painTRAINER interventions is similar. The third group receives a copy of the 2020 edition of the American Chronic Pain Association Resource Guide to Chronic Pain Management [46]. Participants will be followed for 12 months from allocation (Fig. 1).

## Methods: participants, interventions, and outcomes

### Study setting {9}

Participants are being recruited from the populations of 4 integrated healthcare systems which serve as the clinical sites: (1) Kaiser Permanente Georgia (KPGA) with a service area of northern Georgia, (2) Kaiser Permanente Northwest (KPNW) with a service area of Oregon and southwest Washington, (3) Kaiser Permanente Washington (KPWA) with a service area of Washington state, and (4) Essentia Health with services throughout areas of northern Minnesota, eastern North Dakota, and northern Wisconsin. The healthcare facilities in the 3 participating KP regions are located in urban and suburban areas; however, the KP healthcare systems also serve individuals residing in rural areas of their respective regions. Essentia primarily serves rural areas. The RESOLVE study is part of the National Institutes of Health Helping to End Addiction Long-term® (NIH HEAL) Initiative which is an aggressive, trans-NIH effort focused on improving prevention and treatment strategies for opioid misuse and addiction and enhancing pain management. RESOLVE is supported by the HEAL Pain Effectiveness Research Network which is administered by the National Center for Advancing Translational Science (NCATS) Trial Innovation Network [47].

### Eligibility criteria {10}

This study employs a population-based recruitment approach within each of the participating healthcare systems in which the individuals who are invited to be screened for study participation are identified based on the following electronic health records-based criteria.

Electronic health records inclusion criteria:

1. Age 18 years or older
2. Active/enrolled in participating healthcare system for the prior 360 days
3. English speaking and do not need interpreter services
4. Have at least one [at Essentia]* or at least two > 60 days apart [at KP sites] outpatient pain-related healthcare encounters with nonmalignant musculoskeletal pain diagnoses (as determined by ICD10 codes for any of the following: back-pain neck-pain, limb/extremity-pain, joint-pain, arthritic disorders, fibromyalgia, headache, orofacial/ temporomandibular pain, or musculoskeletal pain [48]) within the past 360 days

Electronic health records exclusion criteria:

(5) Have an encounter for surgery related to common musculoskeletal pain conditions (e.g., joint replacement, spinal fusion, carpal tunnel release surgery) [as determined by CPT and/or ICD-10 codes] within the past 60 days
(6) Have two or more separate encounters with a malignant cancer diagnosis other than non-melanoma skin cancer [as determined by ICD-10 codes] within past 60 days
(7) Have ICD-10 code(s), CPT code(s), or department/provider encounters indicating receipt of hospice or other palliative care within the past 360 days
(8) Have ICD-10 codes indicating cognitive impairment severe enough to preclude reasonable participation in a behavioral/ lifestyle change program

*The pain-related healthcare utilization inclusion criteria aim to identify individuals with chronic pain; a unique criterion is used for Essentia because of lower overall in-person utilization rates among the predominately rural population served by this healthcare system.

Individuals who choose to be screened for eligibility must meet the following inclusion and exclusion criteria in order to participate in the study.

Screening inclusion criteria:

1. Have high-impact chronic pain as indicated by self-report endorsing the following 2 items from the Graded Chronic Pain Scale-Revised [49], (1) having pain on “most days” or “every day” in past 3 months (*In the past 3 months, how often did you have pain?* [50]), and (2) pain limiting life or work activities on “most days” or “every day” in the past 3 months (*Over the past 3 months how often did pain limit your life or work activities?* [50])
2. Score of ≥ 12 on the 3-item PEG [range 0–30)] [51, 52] for a recall period of the “past 7 days” [49]

Screening exclusion criteria:

(3) Do not have internet and phone access required for participating in study interventions
(4) Currently receiving, planning to receive in next month, or have received in past 6 months: CBT for pain management, pain management-related psychoeducation, or behavioral skills training for pain management
(5) Currently receiving or planning to receive within the next month: inpatient or intensive outpatient services for substance use disorders
(6) Planning or scheduled to have surgery related to their pain condition within the next 12 months

### Who will take informed consent? {26a}

Participants complete informed consent by phone with a research staff person or electronically via the study website. There are several recruitment and screening steps that precede the informed consent encounter.

Specifically, based on the electronic health records eligibility criteria described above, potentially eligible patients are identified via data query and then mailed a packet containing the study (1) recruitment letter, (2) brochure, and (3) one-page infographic describing the study design and activities. The letter includes instructions for contacting research staff, specific to the potential participants’ clinical site, by phone to address any questions and/or complete eligibility screening by phone. The letter also includes information for accessing the study website and instructions on completing the eligibility screening online, if preferred, including a unique access code for the screening survey. Potential participants who have an active email address and have opted in to research-related communications from their healthcare system (a subset of those mailed) are sent a recruitment email that has similar content to the letter and links to the study website approximately 1 week after the mailing. If a potentially eligible participant does not call the study staff or complete the screening survey within approximately 2 weeks of the mailing, research staff attempt to reach them by phone. The recruitment letter explains this potential outreach and how to opt out of the future contact, if preferred.

If a potential participant is determined to be eligible based on the screening survey, they can proceed with the informed consent process by phone or web during the same encounter, immediately following completion of the screening survey. Or, if the individual would prefer to conduct the informed consent process at a later time, they can schedule a time to complete it by phone with a study staff member or return to the website later to complete it electronically, using their unique code.

#### Informed consent by phone

The research staff member at the clinical site reviews the elements of consent and HIPAA authorization contained in the study information sheet, using the consent phone script. While on the phone, the staff person directs the patient to the study website where the information sheet can be downloaded and reviewed by the patient as the staff person explains/reviews its content. Verbal consent is obtained as well as verbal HIPAA authorization. After completion of the verbal consent process, participants are mailed a copy of the information sheet for their records.

#### Informed consent by web

Individuals determined to be eligible based on the screening survey completed online can continue with the informed consent process by web. They are directed to download and read (via PDF link) the information sheet, which includes all elements of consent and HIPAA authorization. They are also directed to call their site’s study-specific phone line if they have any questions regarding participation. After reading the information sheet, they are asked to electronically record their consent and HIPAA authorization. After completion of the consent process online, participants are mailed a copy of the information sheet for their records.

### Additional consent provisions for collection and use of participant data and biological specimens {26b}

As part of the informed consent process, participants are asked to provide authorization for extraction and use of their healthcare utilization data from their healthcare system’s electronic health record (i.e., encounters with associated pain-related procedures, diagnosis codes, and prescriptions) for the 12 months prior to and following allocation/randomization. These data will be linked to the study-collected assessment data. Participants are also asked to consent to future use of their data as part of the de-identified database stored in the NIH HEAL data repository in accordance with the public data sharing procedures and regulations of the NIH. This trial does not involve collecting biological specimens.

## Interventions

### Explanation for choice of comparators {6b}

The RESOLVE study includes 2 active intervention arms: (1) a health coach-led, 8-session CBT-CP program that is provided one-on-one via telephone or video encounters, and (2) an online, self-directed, 8-session, CBT-CP-based program. Both of these active interventions are compared to (3) an enhanced usual care arm in which participants receive an educational manual, the 2020 edition of the American Chronic Pain Association Resource Guide to Chronic Pain Management [46]. Inclusion of the enhanced usual care comparator enables us to address the critical question: What is the incremental benefit of the study interventions over what treatments patients are already able to access to help manage their pain? This is important to understand from a clinical and healthcare-implementation (cost) perspective. If both interventions perform favorably compared to enhanced usual care, then 2 evidence-based, CBT-CP-based options would be available, providing options for remote care that can be accessed based on factors such as patient preference and healthcare system needs.

This study will also include a comparison of the 2 active, remote interventions. Given the importance of patient motivation and engagement to achieve CBT-CP benefits, we posit that those working with a live therapist via telephone or video may be better able to overcome barriers to routine practice of important skills. This may be particularly important for patients with higher levels of depression or anxiety symptoms (common chronic pain comorbidities) for whom de-activation and/or avoidance may create barriers to implementing effective pain coping skills. Limited data exist on the relative benefits of telephonic/video health coach format versus online delivery format for CBT-CP-based interventions, making this comparison of clinical and cost-effectiveness relative to usual care along with findings from the qualitative evaluation timely and important.

### Intervention description {11a}

Both of the 8-session CBT-CP-based interventions being evaluated in the RESOLVE trial are based on and adapted from the programs developed and refined by Dr. Keefe and colleagues at Duke University over the past three decades [53–58]. In addition, the health coach manual and participant workbook for the RESOLVE health coach-led intervention are based on the PPACT intervention, which was tested in a large pragmatic trial [59, 60]. Table 1 provides an overview of the core content for both of the RESOLVE CBT-CP-based interventions by session. The content and sequencing of the two interventions is consistent and begins with a focus on understanding the neuroscience of pain and the associated rationale for the potential impact of CBT. Sessions focus on learning / mastering the following evidence-based skills: relaxation and body awareness techniques, activity-rest cycling, pleasant activity scheduling, cognitive restructuring, guided imagery, problem solving, and maintenance planning. The RESOLVE interventions are guided by social cognitive theory (e.g., social modeling, vicarious learning) [61, 62], adult learning theory (e.g., tying skills to personal goals and experiences) [63, 64], and behavior change theory [65]. Additionally, the health coach intervention utilizes motivational interviewing techniques to clarify patient values, elicit change talk, and match session focus to patient readiness for change [66] and painTRAINER utilizes the principles of multimedia instruction (interactive exercises, graphics to reinforce explanations) [67]. Participants are considered to have received a full dose of the interventions if at least 75% or any 6 of the 8 sessions are completed.

**Table 1:** Overview and content of RESOLVE interventions by session

Session	Health coach-led telephonic/video conference program	Online program (painTRAINER)
**1**	- Overview of Adaptation Model – Pain Over Time - Overview of Gate Control and Neuromatrix - Introduce and practice **Breath Work**^a^ - Set skills practice goal(s)	- Overview of Pain and its Effects (Adaptation Model) - Overview of Gate Control and Neuromatrix - Introduce and practice **Progressive Muscle Relaxation (PMR)**^a^ - Set skills practice goal(s)
**2**	- Review skills practice from prior week - Introduce and practice **Progressive Muscle Relaxation (PMR)**^a^ - Introduce and practice **Mini-Practices**^a^ - Set skills practice goal(s)	- Review skills practice from prior week - Introduce and practice **Mini-Practices**^a^ - Set skills practice goal(s)
**3**	- Review skills practice from prior week - Introduce **Activity-Rest Cycling**^a^ and how to establish practice - Set skills practice goal(s)	- Review skills practice from prior week - Introduce **Activity-Rest Cycling**^a^ and how to establish practice - Set skills practice goal(s)
**4**	- Review skills practice from prior week - Introduce **Pleasant Activity Scheduling**^a^ and identify / select pleasant activity - Overview of Automatic Thoughts - Introduce A-B-C Model and how to use Thought Records - Set skills practice goal(s)	- Review skills practice from prior week - Introduce **Pleasant Activity Scheduling**^a^ and identify / select pleasant activity - Overview of Automatic Thoughts - Set skills practice goal(s)
**5**	- Review skills practice from prior week - Review A-B-C Model - Introduce and practice developing **Coping Thoughts**^a^ - Set skills practice goal(s)	- Review skills practice from prior week - Overview of Automatic Thoughts - Introduce A-B-C Model and introduce and practice **Coping Thoughts**^a^ - Set skills practice goal(s)
**6**	- Review skills practice from prior week - Introduce and practice **Pleasant Imagery**^a^ - Set skills practice goal(s)	- Review skills practice from prior week - Introduce and practice **Pleasant Imagery**^a^ and **Distraction Techniques**^a^ - Set skills practice goal(s)
**7**	- Review skills practice from prior week - Introduce and practice **Problem Solving**^a^ - Set skills practice goal(s)	- Review skills practice from prior week - Introduce and practice **Problem Solving**^a^ - Set skills practice goal(s)
**8**	- Review of pain coping skills learned in program - Develop a plan for maintaining skills - Celebrate participant milestones	- Review of pain coping skills learned in program - Develop a plan for maintaining skills

^a^Evidence-based pain coping skill

#### Health coach-led program

The health coach-led program includes 8 sessions of CBT-CP provided one-on-one via telephone or video conferencing encounters. The health coaches are required to have at least master’s-level behavioral health training yet are purposefully described as “health coaches” rather than “therapists” to differentiate from mental health therapists and therapy, destigmatizing participation for patients who may otherwise be reluctant to take part. The centralized team of health coaches is based at the KPNW and KPWA clinical study sites. This telehealth model of centralized behavioral health services is being widely adopted across healthcare systems nationally. Each session takes approximately 60 min and provides interactive training in one or more evidence-based pain coping skills. Sessions are scheduled at the participant’s convenience with approximately one session per week and participants have the option of meeting with the health coach by telephone only, using video conferencing or a combination of the two. Participants are asked to complete all 8 sessions within the approximately 12-week post-allocation treatment window. The live health coaching format can enhance patient motivation and engagement by providing patients concrete support in the virtual sessions and helping to tailor skills training approach to patients’ circumstances in real time—elements that may be difficult to fully replicate in self-guided online versions of CBT-CP. The health coaches also utilize motivational interviewing skills to enhance intrinsic motivation to implement recommended skills.

#### Online program

painTRAINER is an online, 8-session, CBT-based program in coping skills training. Each session takes approximately 45 min to complete and provides interactive training in one or more evidence-based pain coping skills [55]. Participants complete sessions on their own, in a set order, and are encouraged to complete one session per week. Research staff assist participants in registering and provide ongoing technical support, but there is no interaction with a trained health coach regarding the treatment content. Session completion is flexible—participants can close a session before completing it and later resume where they left off. They can also go back to review completed sessions or sections of completed sessions (e.g., an audio recording of a skill practice, or instructions on how to use a skill). Participants are asked to complete all 8 sessions within the approximately 12-week post-allocation treatment window. The sessions are led by a recorded “virtual coach” who speaks directly to users. Thus, content is provided in audio to minimize reading and facilitate program completion for low-literacy patients. painTRAINER was designed to include features of therapist-delivered CBT-CP, while providing training in an easy-to-use format that includes animated demonstrations, interactive exercises, tips for working through common barriers to behavior change/skills practice, and tailored feedback to reinforce learning.

### Criteria for discontinuing or modifying allocated interventions {11b}

Any adverse events associated with the active study interventions are expected to be minor, and there are no planned criteria for discontinuing or modifying allocated interventions. There may be rare instances in which an individual decompensates (or is threatening to study staff) and is consequentially unable to continue to actively participate in a behavioral intervention of the type administered in this trial. In this event, the reason for discontinuation will be documented and an attempt to connect the participant to relevant behavioral health resources/support in their healthcare system will be made. If a participant informs the study staff that they will discontinue participation in the intervention, staff will assess their reasons for discontinuation and determine whether the participant wants to continue with the other study activities or withdraw from the study entirely.

### Strategies to improve adherence to interventions {11c}

#### Health coach-led program strategies

##### Training

Treatment fidelity is optimized in this study by the use of a formal structured training process for health coaches that includes approximately 34 h of didactic and experiential coursework using standardized training materials and a standardized treatment manual as well as adequate achievement of proficiency in core skills. The training process included a 90-min overview of pain psychology led by Jennifer Murphy, PhD, study consultant and Frank Keefe, PhD, Co-Investigator. Then for each of the 8 sessions, every health coach completed the following training steps: (1) read content in the treatment manual and participant workbook for the session; (2) participated in a 1-h, web-based, synchronous didactic training for the session; (3) role played as a participant in the session with a clinical supervisor or other health coach; (4) role played the health coach in the session with a clinical supervisor or health coach. Role-played sessions were reviewed in supervision meetings to clarify points and assure adherence prior to moving forward. After these steps were completed, a health coach was assigned their first participant with close monitoring by the clinical supervisors to assess proficiency. Specifically, the clinical supervisors reviewed for proficiency each of the 8 sessions completed with 2 of their initial participants (i.e., 16 sessions total). For each session reviewed, 2 assessments were completed: (1) Fidelity to Session Content and (2) Fidelity to Cognitive Behavioral Therapy Treatment (using a modified version of the VA Cognitive Behavioral Therapy for Chronic Pain Therapist Rating Scale) [19]. A health coach was deemed proficient once they completed at least 2 therapeutic encounters of each of the 8 intervention sessions with 100% fidelity in delivering the session content and 80% fidelity on CBT treatment (i.e., 80% of the 9 domains on the modified Cognitive Behavioral Therapy for Chronic Pain Therapist Rating Scale that are applicable to the session are rated “adequate” or higher). If the first 2 encounters for a session did not meet the above criteria, additional encounters for any deficient session (or sessions) were reviewed until proficiency was met. During this early phase of session review to determine proficiency, written and/or verbal feedback was provided to health coaches on a weekly basis.

##### Ongoing fidelity monitoring

During the study, all sessions with participants are audio- or video-recorded for ongoing supervision and monitoring (unless the participant does not agree to recording the session or there is a technological failure). Health coaches complete a self-assessment of Fidelity of Session Content after each participant session. This self-assessment also includes the health coaches’ clinical notes about the individual components of the session. These self-assessments and notes are reviewed by supervisors as part of the fidelity monitoring process. For the 6 months following when the health coaches reached proficiency, a random sample of 5% of each health coach’s completed sessions were reviewed, using the assessments described above. Once a health coach reached 12 months since the completion of their initial training, 1 session is being randomly selected every 6 months for fidelity review. If a reviewed session does not meet criteria of 100% fidelity in delivering the session content and 80% fidelity on CBT treatment, additional encounters for any deficient session are reviewed until the proficiency criteria are met. Health coaches participate in weekly 1-h supervision meetings. Given there are 12 health coaches, the group was divided into 2 teams to allow sufficient time to review clinical cases. Each meeting consists of research administrative items (e.g., proper documentation, following research procedures) and clinical case reviews. Supervision meetings encourage participation and input from all attendees with a focus on clinical and implementation challenges and solutions as well as success stories.

##### Participant onboarding

Following allocation, participants assigned to receive the health coach-led CBT-CP sessions are mailed materials that include a brief biographical description of their health coach and a participant workbook for the 8 sessions and materials for logging skills practice. The assigned health coach calls the participant approximately 1 week after the materials are mailed. The primary goal of this outreach by the health coach is to introduce themselves and to “onboard” the participant to the program. The onboarding call includes verifying receipt of the packet of materials and orienting the participant to the program structure and format. Participants are informed that coaching calls are confidential and asked their preference for telephone or video coaching calls and for permission to record all coaching calls. The health coach will use the onboarding call as an opportunity to respond to questions from the participant and to schedule the first coaching call. Health coaches follow specified guidelines to complete the onboarding call which include suggested language/scripting.

##### Ongoing participant support

Health coaches work with participants to schedule sessions at the most convenient time for them, with the goal of completing 1 intervention call per week for 8 weeks. Frequently, a standing weekly meeting time is identified for the 8 weeks; however, there is flexibility in this schedule and health coaches can accommodate scheduling challenges, as necessary. Health coaches may remind participants 1–2 days before a scheduled session via phone or email (based on participant preference). If a participant does not attend a scheduled call, the health coach will first reach out by telephone to reconnect and reschedule the missed appointment. When reached, the health coach may explore possible barriers to attending sessions and work with the participant to problem-solve for any challenges, as appropriate. If the participant is not reached right away, multiple outreach attempts will be made over the course of several weeks, using different contact modes, including phone, email and mailed letters, and text messaging.

#### Online program strategies

##### Fidelity monitoring

Treatment is standardized because the intervention is delivered via an online program; thus, no fidelity monitoring is possible nor warranted.

##### Participant onboarding and registration

Following allocation, participants who are assigned to receive the painTRAINER online program are mailed materials that include instructions for registering for the program, unique login information, a participant workbook, and study team contact information (in case of questions or technical difficulties). Approximately 1 week after the materials are mailed, study staff call participants to “onboard” them to the program. This entails providing a verbal overview of the program and making sure participants register and can access/log into painTRAINER. Study staff follow specified guidelines to complete the onboarding call which include suggested language/scripting that has MI-based language [66]. The primary goal of the onboarding call is to ensure that participants register and have no technical difficulties. The inclusion of MI-based language allows the staff person doing the onboarding to have participants consider potential barriers to participation and how they might plan to overcome such challenges, should they arise, as well as to support and encourage their commitment to utilize painTRAINER to learn skills to manage their pain.

##### Ongoing participant support

After participants begin the painTRAINER program, automated emails are sent about once per week to remind participants to complete the painTRAINER sessions but can be turned off by the participant. In addition, automated reminders to practice skills can be set by the participant to occur daily, weekly, or not at all, depending on their preference. Support is also provided by study staff if a participant is not adhering to the recommended completion schedule. Specifically, a study staff person will follow-up with a participant by phone in the following circumstances:

- It has been more than 7 days since the participant registered and session 1 has not been initiated.
- A session has been initiated but is still incomplete after more than 7 days.
- It has been more than 10 days since the most recent session completion date and another session has not been initiated.
- Sessions are being completed too quickly (3 or more sessions completed in 9 days).

Study staff utilize specified guidelines to complete follow-up calls which include suggested language/scripting. The goal of the follow-up call is to identify barriers to participation and ways of overcoming such barriers (or addressing technical needs), thereby encouraging ongoing adherence.

### Relevant concomitant care permitted or prohibited during the trial {11d}

Eligibility screening criteria exclude from participation individuals who are currently receiving, are planning to receive in the next month, or have received in past 6 months CBT for pain or a similar psychoeducational skill-based training for pain. However, once enrolled, participants in all study groups can receive any pharmacologic or nonpharmacologic treatments available to them without restriction. At each follow-up study assessment (T_1_, T_2_, T_3_), participants’ use of CBT for pain or a similar psychoeducational skill-based training for pain (in-person, by phone or via online or App-based programs) will be assessed. Receipt of opioids in a manner consistent with long-term opioid treatment is also tracked via electronic health records data.

### Provisions for post-trial care {30}

Post-trial care will not be provided. There is no anticipated harm for trial participation and therefore no compensation for anticipated harm.

### Outcomes {12}

Table 2 provides information on primary and secondary study objectives and outcomes. The RESOLVE study employs the NIH HEAL Common Data Elements [68] and additional select outcomes.

**Table 2:** Primary and secondary outcomes overview

Objective	Outcome			Time frame (s)
	**Description**	**Source**	**Type**	
**To determine whether the active interventions result in a higher proportion of patients achieving a reduction in *pain severity* that is a minimal clinically important difference (MCID) relative to those receiving usual care at 3 months (*T***_**1**_**) post-allocation**	≥ 30% decrease in score on 11-item version of the Brief Pain Inventory – Short Form (BPI-SF) [69–71]	Participant self-report	Binary	*T*_0_ to *T*_1_
Secondary Objective 1 To determine whether the active interventions result in a higher proportion of patients achieving a MCID in *pain severity* relative to those receiving usual care at 6 (*T*_2_) and 12 months (*T*_3_) post-allocation	Same as above	Participant self-report	Binary	*T*_0_ to *T*_2_ *T*_0_ to *T*_3_
Secondary Objective 2 To determine whether the active interventions result in a higher proportion of patients achieving a MCID in *pain intensity* relative to those receiving usual care at 3 (*T*_1_), 6 (*T*_2_), and 12 months (*T*_3_) post-allocation	≥ 30% decrease in score on 4-item pain intensity subscale of the BPI-SF	Participant self-report	Binary	*T*_0_ to *T*_1_ *T*_0_ to *T*_2_ *T*_0_ to *T*_3_
Secondary Objective 3 To determine whether the active interventions result in a higher proportion of patients achieving a MCID in *pain-related interference* relative to those receiving usual care at 3 (*T*_1_), 6 (*T*_2_), and 12 months (*T*_3_) post-allocation	≥ 30% decrease in score on 7-item pain-related interference subscale of the BPI-SF	Participant self-report	Binary	*T*_0_ to *T*_1_ *T*_0_ to *T*_2_ *T*_0_ to *T*_3_
Secondary Objective 4 To examine the impact of the active interventions on *pain severity* at 3 (*T*_1_), 6 (*T*_2_), and 12 months (*T*_3_) post-allocation	Score on modified 11-item BPI-SF	Participant self-report	Continuous	*T*_0_*, T*_1_*, T*_2_, and *T*_3_
Secondary Objective 5 To examine the impact of the active interventions on *pain intensity* at 3 (*T*_1_), 6 (*T*_2_), and 12 months (*T*_3_) post-allocation	Score on 4-item pain intensity subscale of the BPI-SF	Participant self-report	Continuous	*T*_0_*, T*_1_*, T*_2_, and *T*_3_
Secondary Objective 6 To examine the impact of the active interventions on *pain-related interference* at 3 (*T*_1_), 6 (*T*_2_), and 12 months (*T*_3_) post-allocation	Score on 7-item pain-related interference subscale of the BPI-SF	Participant self-report	Continuous	*T*_0_*, T*_1_*, T*_2_, and *T*_3_
Secondary Objective 7 To examine the impact of the active interventions on *social role functioning* at 3 (*T*_1_), 6 (*T*_2_), and 12 months (*T*_3_) post-allocation	PROMIS Ability to Participate in Social Roles 4A [72] (4 items)	Participant self-report	Continuous	*T*_0_*, T*_1_*, T*_2_*,* and *T*_3_
Secondary Objective 8 To examine the impact of the active interventions on *physical functioning* at 3 (*T*_1_), 6 (*T*_2_), and 12 months (*T*_3_) post-allocation	PROMIS Physical Functioning Short Form 6b (6 items)	Participant self-report	Continuous	*T*_0_*, T*_1_*, T*_2_, and *T*_3_
Secondary Objective 9 To examine the impact of the active interventions on patient global impression of change (PGIC) in *pain status* and *overall status*	Modified Guy/Farrar Patient Global Impression of Change (1 item for pain status and 1 item for overall status)	Participant self-report	Continuous	*T*_0_*, T*_1_*, T*_2_, and *T*_3_
**To assess the costs and cost-effectiveness of the active interventions compared to each other and usual care**	EuroQuol-5D-5L [73] to construct cost per quality-adjusted life year (QALY) Healthcare utilization: Electronic health records data costed using standard costing algorithms [74, 75] and Medicare fee schedules	Participant self-report and electronic health records	Continuous	*T*_0_,*T*_1_, T_2_ and *T*_3_ *T*_0_ minus 12 months

#### Primary outcome

The primary outcome measure is a minimal clinically important difference (MCID) in pain severity (composite of pain intensity and pain-related interference). MCID is determined as a 30% decrease in score on a modified 11-item version of the Brief Pain Inventory – Short Form (BPI-SF) [69–71] from allocation (T_0_) to 3 months post-allocation (T_1_). The choice of primary outcome measure is consistent with IMMPACT consensus guidelines [76]. The BPI-SF consists of 15 items and assesses the following areas: severity of pain, intensity of pain, impact of pain on daily function, location of pain, pain medications, and amount of pain relief in the past 24 h or the past 7 days [69–71]. The RESOLVE trial utilizes an 11-item modified version of the BPI-SF that excludes items assessing location of pain, pain medications, and amount of pain relief from treatments/medications and employs the past 7-day assessment time frame (versus past 24 h). The 11 items included in the modified BPI-SF and the corresponding 11-point Likert scale for each are provided below. Pain severity score is the calculated mean of all 11 items; range 0–10 with a higher score = worse pain severity [49, 77]. (Note: Items 1–4 comprise the pain intensity subscale and items 5–11 comprise the pain-related interference subscale). All 4 items of the pain intensity subscale items and at least 4 out of the 7 pain-related interference subscale items must be complete to score pain severity.

1. *What number best describes ****your pain at its worst**** in the past 7 days?* [0 = No pain, 1, 2, 3, 4, 5, 6, 78, 8, 9, 10 = Pain as bad as you can imagine]
2. *What number best describes ****your pain at its least**** in the past 7 days?* [0 = No pain, 1, 2, 3, 4, 5, 6, 78, 8, 9, 10 = Pain as bad as you can imagine]
3. *What number best describes ****your pain on average**** in the past 7 days?* [0 = No pain, 1, 2, 3, 4, 5, 6, 78, 8, 9, 10 = Pain as bad as you can imagine]
4. *What number best describes how much ****pain you have right now?*** [0 = No pain, 1, 2, 3, 4, 5, 6, 78, 8, 9, 10 = Pain as bad as you can imagine]
5. *What number best describes how pain has interfered with your ****general activity**** during the past 7 days?* [0 = No interference, 1, 2, 3, 4, 5, 6, 78, 8, 9, 10 = Complete interference]
6. *What number best describes how pain has interfered with your ****mood**** during the past 7 days?* [0 = No interference, 1, 2, 3, 4, 5, 6, 78, 8, 9, 10 = Complete interference]
7. *What number best describes how pain has interfered with your ****walking ability**** during the past 7 days?* [0 = No interference, 1, 2, 3, 4, 5, 6, 78, 8, 9, 10 = Complete interference]
8. *What number best describes how pain has interfered with your ****normal work, including both work outside the home and housework,**** during the past 7 days?* [0 = No interference, 1, 2, 3, 4, 5, 6, 78, 8, 9, 10 = Complete interference]
9. *What number best describes how pain has interfered with your ****relations with other people**** during the past 7 days?* [0 = No interference, 1, 2, 3, 4, 5, 6, 78, 8, 9, 10 = Complete interference]
10. *What number best describes how pain has interfered with your ****sleep**** during the past 7 days?* [0 = No interference, 1, 2, 3, 4, 5, 6, 78, 8, 9, 10 = Complete interference]
11. *What number best describes how pain has interfered with your ****enjoyment of life**** during the past 7 days?* [0 = No interference, 1, 2, 3, 4, 5, 6, 78, 8, 9, 10 = Complete interference]

#### Secondary outcome measurements

##### Pain outcomes

Secondary pain outcomes include MCID in pain severity (as described above) from allocation (T_0_) to 6 months (T_2_) and 12 months (T_3_) post-allocation. In addition, MCID for the 4-item pain intensity subscale and the 7-item pain-related interference subscale of the BPI-SF composite measure will be assessed from allocation (T_0_) to each follow-up time point: 3 months (T_1_), 6 months (T_2_), and 12 months (T_3_) post-allocation. Lastly, change in score from allocation (T_0_) for pain severity, pain intensity, and pain-related interference (continuous) will be assessed at T_1_, T_2_, and T_3_.

##### Quality of life-related outcomes

Chronic pain has a profound effect on overall quality of life and the process by which pain affects emotional well-being involves both disrupted physical and social functioning with the disruption of social relationships seeming the larger contributor of the two [78]. Social role functioning will be assessed using the Patient-Reported Outcomes Measurement Information System (PROMIS) Ability to Participate in Social Roles 4A [72]. It is a 4-item assessment that generates a continuous score with a range of 4 to 20. A higher score indicates a better ability to participate in social roles. Physical functioning will be assessed using the PROMIS Physical Functioning Short Form 6b. It is a 6-item assessment that generates a continuous score with a range of 0 to 6. A higher score indicates a better physical functioning.

Patient global impression of change (PGIC) will be assessed in two areas: (1) overall pain and (2) overall status using a modified 7-point PGIC scale (also referred to as the original Guy/Farrar-PGIC scale). The PGIC scale is used to measure global treatment effect and is recommended as a compliment to pain severity scales. Although other versions of the PGIC scale exist, the original Guy/Farrar-PGIC scale is recommended for clinical trials since it has been used extensively and shown to be sensitive to change [79, 80]. The original Guy/Farrar-PGIC scale uses the following response options: 0—Very much improved, 1—Much improved, 2—Minimally improved, 3—No change, 4—Minimally worse, 5—Much worse, 6—Very much worse. The modified version used in this trial employs the following response options as has been done in multiple large pain management trials (SCOPE [81], CAMMPS [82], ESCAPE [37], SPACE [83, 84]): 0—Much better, 1—Moderately better, 2—A little better, 3—No change, 4—A little worse, 5—Moderately worse, 6—Much worse.

##### Moderators

Subgroup analyses will be conducted to determine the impact of the active interventions on specific populations and explore for potential heterogeneity of treatment effects by sex, age, race/ethnicity, rural/medically underserved residency, multiple pain conditions, mental health disorders, and negative social determinants of health. Table 3 describes these variables.

**Table 3:** Moderator variables for subgroup analyses

Moderator	Definition	Data source
Sex	Male vs. Female/Other	Patient self-report at *T*_0_
Age	< 65 vs. ≥ 65 years old	Electronic health records at *T*_0_
Race/ethnicity	White/Non-Hispanic Black or African American/Non-Hispanic, Hispanic Other	Patient self-report at *T*_0_
Rural/medically underserved residency	Urban vs. rural or medically underserved Rural is defined as subject’s resident Census Tract corresponds to US Census 2010 Rural–Urban Commuting Area (RUCA) Codes 4, 5, 6, 7, 8, 9, or 10 [85] Medically underserved is defined as subject’s resident Census Tract corresponds to HRSA-designated primary care or mental health geographic or geographic high needs health professional shortage area [86]	Electronic health records geocoded data at *T*_0_
Multiple nonmalignant musculoskeletal pain conditions	1 pain cluster vs. > 1 pain cluster Cluster based on ICD-10 diagnoses corresponding with nonmalignant musculoskeletal chronic pain condition developed for the National Pain Strategy chronic pain condition clusters [48]	Electronic health records data at *T*_0_minus 360 days
Mental health disorders	ICD-10 diagnosis for depression and/or anxiety	Electronic health records data at *T*_0_ minus 360 days
Negative social determinants of health (SDH)	Negative SDH/existing need vs. No SDH need Patient endorses need in one or more of the following domains: (1) Financial Resource Strain (1 item) (2) Food Insecurity (2 items) (3) Transportation/Access Needs (2 items) (4) Housing Instability (3 items)	Patient self-report at *T*_0_

##### Economic outcomes

The EQ-5D-5L is a validated measure of health-related quality of life, designed to estimate quality-adjusted life years (QALYs) [87]. It is widely used in economic evaluations across different disease areas. It contains five questions that are related to a different domain of everyday life: (1) mobility, (2) self-care, (3) usual activities, (4) pain/discomfort, and (5) anxiety/depression. For each domain, the respondent indicates whether they experience no problems, slight problems, moderate problems, severe problems, or extreme problems. The EQ-5D-5L also includes a visual analog scale for participants to rate their overall health on a scale from 0 (“worst imaginable health”) to 100 (“best imaginable health”). An individual’s responses are converted to a single summary utility score using region-specific value sets; the current study will use U.S.-based values [88], which include values less than zero and represent states of health considered worse than death. Using the framework of cost-effectiveness, the incremental cost per additional patient with a MCID in pain severity (30% reduction from baseline) and the incremental cost per QALY gained will be estimated separately at 12 months. Gains in QALYs will be calculated using by assessing changes in utility scores derived from the EQ-5D-5L across 12 months of follow-up [87].

Costs based on healthcare utilization and intervention costs will be assessed. Healthcare utilization will be generated using electronic health records and administrative data and costed using standard costing algorithms [74, 75] and Medicare fee schedules. Intervention costs will be calculated using process data related to all relevant resources used in the intervention delivery.

##### Exploratory outcomes

Additional exploratory outcomes for quantitative analysis include long-term opioid use, comorbid symptomology, high-impact chronic pain, and chronic pain grade as described in Table 4.

**Table 4:** Exploratory outcomes

Outcome	Description	Data source	Time frame
Long-term opioid use	Opioid prescription orders or fills indicating a continuous ≥ 60-day supply during the prior 90-day period (Binary)	Electronic health record	*T*_0_*, T*_1_*, T*_2_*,* and *T*_3_
Depression symptomology	Patient Health Questionnaire-8 (PHQ-8) [89] (8 items; continuous)	Patient self-report	*T*_0_, *T*_1_, *T*_2_, and *T*_3_
Anxiety symptomology	Generalized Anxiety Disorder-7 (GAD-7) [90] (7 items; continuous)	Patient self-report	*T*_0_, *T*_1_, *T*_2_, and T_3_
Sleep disturbance	PROMIS Sleep Disturbance – Short Form 6a [91] (6 items; continuous)	Patient self-report	*T*_0_, *T*_1_, *T*_2_, and *T*_3_
High-impact chronic pain	High-Impact Chronic Pain [3, 49]	Patient self-report	*T*_0_, *T*_1_, *T*_2_, and *T*_3_
Graded chronic pain	Graded Chronic Pain Scale-Revised [49]	Patient self-report	*T*_0_, *T*_1_, *T*_2_, and *T*_3_

In addition, the following variables will be assessed as mediators: pain catastrophizing and pain self-efficacy assessed at T_0_ and T_1_ using the Pain Catastrophizing Scale-Short Form 6 [92] and Self-Efficacy for Pain Management subscale of the Chronic Pain Self-Efficacy Scale [93]; perceived support from study intervention assessed at the time of notification of randomization group (2 items developed by study team).

### Process evaluation

The RESOLVE study includes a mixed-method evaluation to understand (1) patient experiences of the interventions, including how they relate to treatment response, clinical site, and rural/medically underserved residency status; and (2) healthcare system issues, including adaptations and contextual factors at the site and external levels, barriers and facilitators to intervention success, and potential for adoption, sustainability, and dissemination. As part of the mixed-method evaluation, interview data will be collected at a range of timepoints and reflective of multiple perspectives, including active intervention participants, participants that drop out or disengage from the interventions; health coaches, staff involved in onboarding participants to the online program; and healthcare system leaders at each site who are involved in managing services for chronic pain patients. Table 5 summarizes the interviews to be conducted.

**Table 5:** Overview of qualitative interviews

Stakeholder group	Time period for interviews	Number of interviewees	Focus of interviews
Active participants in both health coach-led and online interventions	Twice: 3–5 months post-randomization (*T*_0_) and 12–14 months post-randomization (*T*_3_)	70–80 participants balanced on intervention group, site, age, gender, urban/rural location, and race/ethnicity	- Motivations/expectations - Barriers/facilitators to engagement - Barriers/facilitators to skill practice and maintenance over time - Sense of perceived helpfulness for pain management or other benefits - Areas for improvement
Participants who disengage	One interview after drop or disengagement from intervention	20 participants balanced on intervention group	- Motivations/expectations - Barriers/facilitators to engagement - Sense of perceived helpfulness or not - Reasons for disengagement - Areas for improvement
Health coaches	Twice: first approximately 6 months after starting and second approximately 1 year later	Approximately 11–12 health coaches each time	- Reflections on training and preparation for delivering intervention - Barriers/facilitators to reaching and engaging participants - Barriers/facilitators to teaching content and skills - Areas for improvements
Staff supporting online program	Twice: first about 6 months of intervention start and second approximately 1 year later	Approximately 4–6 staff each time	- Reflections on onboarding and supporting participants in signing up for online program - Barriers/facilitators to reaching and engaging participants - Areas for improvements
Healthcare leaders at participating sites	Up to 2 times: first towards end of intervention period and second approximately 6 months later	Approximately 8–10 (1 to 2 per site)	- Reactions and reflections on the two intervention groups - Current and proposed services being offered to chronic pain patients - Suggestions for adaptations for the two interventions - Barriers/facilitators to maintenance and integration of interventions - Areas for improvements

Active intervention participants will be interviewed twice after completion of the 8-session intervention, with the first interview occurring post-treatment (approximately 3 months post-allocation; T_1_) and the second interview occurring 9 months post-treatment (approximately 12 months post-allocation; T_3_). We will explore barriers to and facilitators of engaging in the two interventions and learning and practicing the skills, as well as willingness and ability to utilize and maintain the skills over time. We will also assess perceived benefits and/or negative consequences on pain, pain management, and other possible outcomes (e.g., improved sleep). We aim to interview 70–80 participants, balanced by intervention group, site, age, rural/urban residency, pain severity score at T_1_, and representative of race/ethnicity. This number of interviews is sufficient for meeting saturation for content analysis and identifying themes [94]. Additionally, we will interview 20 participants who disengage from the interventions early on to assess barriers to participation.

Health coaches (*n* = 12) who deliver the remote CBT-CP intervention will be interviewed twice, approximately 6 months after they begin treating patients in the study and again approximately 12 months later. These interviews will explore health coach perspectives on challenges of and successes to delivering the intervention over time and will identify suggestions for improvement and future implementation of remote CBT-CP interventions. We will conduct similar interviews with staff who onboard participants to the painTRAINER program. In addition, healthcare leaders at each participating site (8–10 total) involved in managing or delivering care for chronic pain patients will be identified and invited for 1 to 2 interviews to explore their reactions to the 2 interventions and explore possible areas of adaptation and maintenance for integrating the interventions at their sites.

All interview data will be managed by an experienced and trained qualitative team led by a doctoral trained medical anthropologist (Carmit McMullen, Co-Investigator). Interviews will be conducted by phone, recorded on password-protected devices, and professionally transcribed to aid in content analysis. An inductive, constant comparative approach [95] will be used to summarize interview data. Mixed method data management and coding/analysis of interview data will be aided by the qualitative software program NVivo R1.6 [96].

The process evaluation is informed by 2 frameworks which guide data collection and analysis. First is the RE-AIM model [97, 98], which has 4 components: Reach, Effectiveness, Adoption, Implementation, and Maintenance. Electronic health records, interview, participant tracking and survey data will contribute to understanding RE-AIM components. Reach reflects the percentage and characteristics of persons who receive or are affected by a program. The project will use electronic health records and study tracking data to examine (1) the percentage of patients excluded from the trial and the rationale for exclusion, and (2) the percentage of patients who participate in the program based on the denominator of all patients who were approached for participation in each healthcare system, as well as all potentially eligible patients in the healthcare system regardless of whether or not they were approached for participation. Effectiveness measures the impact of the intervention on primary and secondary outcomes. Trial outcomes and participant interview data will be used to assess and explain effectiveness. Adoption is less relevant to the current study as the telehealth programs can and will be made available to patients completely outside the ambulatory care setting, thereby reducing or eliminating routine barriers for adoption of healthcare treatments. However, health system leader interviews will provide insight about potential for future adoption. Implementation will be assessed by examining participant completion of the 2 interventions (data recorded by the health coaches and automated data collected from the painTRAINER platform). Interview data will identify barriers and facilitators to engagement and completion of intervention activities. Finally, maintenance of skills learned will be assessed through interviews with participants immediately following and 9 months after intervention completion. In addition to RE-AIM, the Theoretical Framework of Acceptability (TFA) will be used to explore various aspects of acceptability, from both participant and healthcare system perspectives [99]. The TFA is a multifaceted construct that reflects the extent to which people delivering or receiving a healthcare intervention consider it to be appropriate. The TFA is complimentary to constructs in RE-AIM and further examines issues of acceptability, particularly for patients. The TFA consists of 8 primary domains that have temporal aspects (e.g., before, during, and after an intervention) and consider both anticipated reactions to an intervention as well as cognitive and emotional responses experienced with an intervention. The domains include affective attitude, burden, ethicality, intervention coherence, opportunity costs, perceived effectiveness, and self-efficacy.

### Participant timeline {13}

**Fig. 2:**
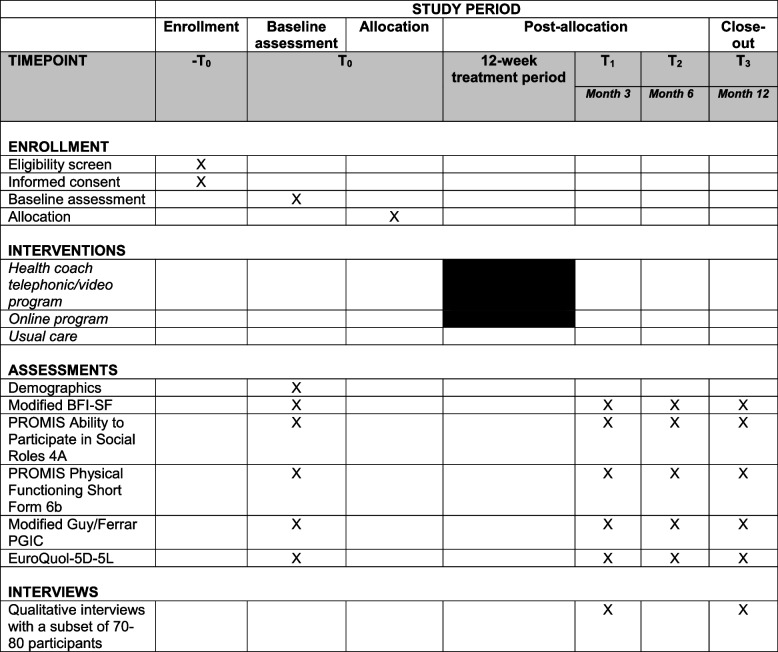
RESOLVE schedule of enrollment, interventions, and assessments for participants

See Fig. 2 for a schedule of participant activities.

### Sample size {14}

For the primary study outcome, MCID in pain severity from T_0_ to T_1_, sample size requirements were calculated for a two-sided comparison of independent proportions with 90% power using Fisher’s least significant difference method to account for three-way comparisons (specifically conduct Omnibus Wald test for any difference between three groups and then conduct pairwise comparisons given an overall difference is statistically significant at the 0.05 alpha level). Sample size calculations assumed a usual care outcome rate of 15% who achieve the MCID of ≥ 30% reduction in pain severity from T_0_ to T_1_. The 15% usual care rate was chosen because it was observed among participants in the usual care arm of the study principal investigator’s recently completed PPACT trial [24]. The necessary analytic sample size of 1863 (621 per arm) was calculated to detect a difference of 7.5% between a given intervention group relative to usual care in the proportion of individuals who attain a clinically meaningful change in pain severity. A retention rate of 80% is estimated. Thus, to achieve this final analytic sample size, at least 2331 individuals will be randomized, approximately 777 per intervention arm. A 7.5% detectable difference corresponds to 22.5% (relative change of 150%) of individuals in an intervention arm attaining a MCID in pain severity.

Power for secondary, subgroup analyses was also estimated (Table 6). Sample size requirements for these secondary analyses were calculated using the same assumptions described above (i.e., a two-sided comparison of independent proportions using Fisher’s least significant difference approach to account for three-way comparisons, with sample size calculations assuming an improvement rate of 15% in the usual care arm). The subgroup sample size was projected to range between 20 and 40% of the original 1863 sample size since this should cover the range of the subgroup sample sizes of interest.

**Table 6:** Power for subgroup analyses ranging the size of the subgroup

Assumptions		80% power to detect:		
**Subgroup sample size**	**Usual care (UC)**	**Intervention (Int)**	**Detectable difference**	**Relative change**
***N* (% of 1863)**	**% UC**	**% Int**	**%Int—%UC**	**% Int / %UC**
372 (20%)	15.0%	31.0%	16.0%	206.7%
558 (30%)	15.0%	27.7%	12.7%	184.7%
744 (40%)	15.0%	25.8%	10.8%	172.0%

There is 80% power to detect a 10.8% difference (relative change of 172%) between each intervention group and usual care if subgroup sample size is 40% of the overall study population and 16.0% difference (relative change of 206.7%) if the subgroup sample size is 20% of the overall study population. Note that in previous studies of this study team, sex and comorbid mental health conditions (depression, anxiety) were not less than 40% of the population suggesting there is high power for the primary subgroups of interest. Further, the study is aiming to have at least 20% of rural/medically underserved residency and therefore there is good power for this subgroup. Individuals with negative social determinants of health comprise an exploratory subgroup since it is not clear how many people in this population a priori will have this indication.

### Recruitment {15}

Each of the 4 sites is responsible for recruiting and enrolling approximately 600 patients per site in order to reach the overall study target for number randomized within the study timeline (*N* = 2331). On a monthly basis during the approximately 24-month recruitment period, each site queries their electronic health records data warehouses to identify a random sample of patients who meet the electronic health record-based inclusion criteria. The sample of patients is stratified by urban vs. rural or medically underserved residence, based on the individuals’ resident Census Tract/geocoded data [85, 86].

As described previously in the section related to informed consent, individuals in the random sample are mailed a recruitment letter and brochure which includes a description of the study, instructions for how to call a research staff person to complete the screening survey by phone or complete it online, and select elements of informed consent, including a clear statement of the ability to opt out of further contact by calling the site-specific study telephone number. Finally, the letter states that patients might be contacted by phone in the following weeks to participate in the study if they have not called to opt out of further contact or gone online to complete the screening survey. Each site, as feasible/allowable, will also send a recruitment email in follow-up to the mailing to patients in the sample who have an active email address.

This population-based recruitment process is the primary method for identifying potentially eligible patients for this trial. A secondary method is self-referral. Individuals who learn about the study on their own (not through the targeted mailing) can contact their site. Study staff will obtain their permission to access their electronic health record, and then the site analyst will execute a pre-developed program to evaluate eligibility. If the person does not meet the electronic health record inclusion criteria, the study staff person will follow-up with the patient by phone or mailed letter to explain this. If the person does meet the electronic health record inclusion criteria, they will be mailed recruitment materials and follow the same process as described above for those identified through the monthly queries.

## Assignment of interventions: allocation

### Sequence generation {16a}

After completion of the baseline assessment, participants are individually randomized in equal ratio to one of the three study groups: (1) telephonic/video conference program, (2) online program, or (3) usual care plus manual. Randomization is stratified on sex as documented in the electronic health record (male vs. female or other); pain severity score on 11-item modified BPI-SF from baseline assessment (< 7 vs. ≥ 7); clinical recruitment site (KPGA, KPNW, KPWA, Essentia Health); and residency in a rural or medically underserved area based on residency geocode for Census Tract from electronic health record (yes vs. no). A random permutated block design is used with random variable block sizes of 3, 6, or 9 to ensure approximately equal accrual over time into the three study groups.

### Concealment mechanism {16b}

Participants are randomized within the study’s secure, web-based, electronic data capture system immediately after participant completion of the baseline assessment. The lead biostatistician at the NIH HEAL Johns Hopkins University Trial Innovation Center Biostatistics Core, in collaboration with the study biostatistician (Andrea Cook), developed the randomization scheme. The Utah Data Coordinating Center developed the randomization tables for integration into the electronic data capture system.

### Implementation {16c}

Following randomization within the electronic data capture system (EDC), participants appear on cohort management lists and sections of the EDC that are applicable to intervention delivery for logging intervention contacts and session completion and are accessible only by unblinded staff (i.e., research support staff who mail intervention materials, health coaches). Information on the allocation group will remain concealed to study outcome assessment staff. Participants receive a mailing within approximately 1 week of allocation that indicates the study group to which they have been assigned and includes any relevant materials related to their group.

## Assignment of interventions: blinding

### Who will be blinded {17a}

The outcome assessors are blinded to group allocation. During assessments, participants are instructed to not communicate with the assessors about the intervention received.

In addition, all study team investigators will remain blinded and not have access to outcome data or results until the completion of the final 12 months outcome assessments in which the datasets will be locked. After the 3-month outcome data collection is complete, the independent Data Coordinating Center at the University of Utah, independent Biostatistical Core at John Hopkins University, and the study team’s collaborative biostatistician will have access to outcome data without treatment allocation for programming purposes. The study biostatistician Dr. Cook, will not have access to data or see any results, remaining blinded until after the 12-month outcome assessments are complete and final datasets are locked. The statistical analyses will be conducted blind to actual randomization assignment until final.

### Procedure for unblinding if needed {17b}

The outcome assessors will not be unblinded during the trial. There are no planned interim analyses so unblinding of statisticians is not anticipated prior to the data lock unless requested by the independent Data Safety and Monitoring Board.

## Data collection and management

### Plans for assessment and collection of outcomes {18a}

Assessments will be completed via questionnaires administered at baseline (T_0_), 3 months post-randomization (T_1_; post-treatment), 6 months post-randomization (T_2_), and 12 months post-randomization (T_3_). The research team will keep careful track of assessment completion and will contact participants (by phone, email, mail) if measures have not been completed according to a protocol to encourage completion of assessments at all timepoints.

### Plans to promote participant retention and complete follow-up {18b}

The following strategies will be used to maximize follow-up assessment completion rates: (1) allow participants to complete assessments using the mode they prefer (online, by telephone, or a mailed survey) given reasonable comparability expected across modalities of administration [100, 101]; (2) confirm and update participant contact information (phone, address, email) at each contact; (3) use healthcare system administrative records to identify any address changes that are not communicated by the participant; and (4) provide incentives that adequately compensate subjects for the time spent completing assessments.

### Data management {19}

Follow-up assessment data are stored on a HIPAA-compliant secure server hosted, managed, and monitored by the University of Utah Data Coordinating Center (DCC), with daily backups, and will be de-identified at the earliest possible opportunity. The DCC oversees data management for the trial and details on their processes and procedures are specified in the Data Management Plan which is available from the corresponding author on request.

### Confidentiality {27}

Subject confidentiality is strictly held in trust by the investigators, study staff, and the study sponsor(s) and their agents. This confidentiality is extended to any study information relating to participants. The study protocol, documentation, data, and all other information generated will be held in strict confidence. No information concerning the study or data will be released to any unauthorized third party without prior written approval of the study sponsor. The U.S. Department of Health and Human Services (HHS) has issued a Certificate of Confidentiality and, as a recipient of NIH funding for human subjects research, this study complies with the requirements for protection of identifiable research information from forced disclosure per the terms of the NIH Policy. As a recipient conducting NIH-supported research covered by this Policy, the study team has established and maintains internal policies and procedures to ensure management in compliance with Federal statutes, regulations, and the terms and conditions of the award.

### Plans for collection, laboratory evaluation, and storage of biological specimens for genetic or molecular analysis in this trial/future use {33}

This trial will not involve the collection of biological specimens.

## Statistical methods

### Statistical methods for primary and secondary outcomes {20a}

We will use modified Poisson regression [102] fit using generalized estimating equations (GEE) to model the binary primary outcome, MCID in pain severity (30% reduction from baseline), 3 months (T_1_; primary timepoint) and 6 and 12 months (T_2_ and T_3_; secondary timepoints). We are employing modified Poisson (i.e., Poisson family with log link, but use robust standard errors to correct for mis-specified outcome variance) instead of logistic regression since the binary outcome is not rare and the estimate of interest is the relative risk. We will use a working independence correlation matrix and will calculate standard errors using the robust sandwich estimator to account for within-person and within-health coach correlation [103] and account for the mis-specified mean–variance structure when using Poisson regression for a binary outcome [102, 104]. We will include interactions between each intervention and indicators of time (T_2_ and T_3_) to estimate time-specific intervention effects; the primary comparisons will be between the interventions and usual care at 3 months (i.e., primary effectiveness will test the size of the intervention coefficient at the 3-month timepoint). We will adjust for baseline levels of pain severity, other stratification variables (sex, clinical site, and rural/medically underserved residency), and a priori variables predictive of outcome (multisite pain and co-occurring mental health condition). Specifically, we will fit the following mean model where usual care and 3 months are the reference groups:

$$\mathrm{log}\left(\mathrm{E}({\mathrm{Y}}_{\mathrm{ij}})\right)={\upbeta }_{\mathrm{o}}+{\upbeta }_{1}{\mathrm{Int}1}_{\mathrm{i}}+{\upbeta }_{2}{\mathrm{Int}2}_{\mathrm{i}}+{\upbeta }_{3}{\mathrm{T}1}_{\mathrm{ij}}+{\upbeta }_{4}{\mathrm{T}2}_{\mathrm{ij}}+{\upbeta }_{5}{\mathrm{Int}1}_{\mathrm{i}}{\mathrm{T}1}_{\mathrm{ij}}+{\upbeta }_{6}{\mathrm{Int}2}_{\mathrm{i}}{\mathrm{T}1}_{\mathrm{ij}}+{\upbeta }_{7}{\mathrm{Int}1}_{\mathrm{i}}{\mathrm{T}2}_{\mathrm{ij}}+{\upbeta }_{8}{\mathrm{Int}2}_{\mathrm{i}}{\mathrm{T}2}_{\mathrm{ij}}+{\upbeta }_{\mathrm{z}}{\mathrm{Z}}_{\mathrm{i}}$$

where ${\mathrm{Y}}_{\mathrm{ij}}$ is the binary MCID outcome for participant i (i = 1,…,n) and timepoint j (j = 1,2,3), ${\mathrm{Int}1}_{\mathrm{i}}$ is 1 if the participant i is randomized to the first intervention group and 0 otherwise, ${\mathrm{Int}2}_{\mathrm{i}}$ is 1 if participant i is randomized to the second intervention group and 0 otherwise, ${\mathrm{T}1}_{\mathrm{ij}}$ is 1 if outcome is measured for participant i at 6 months (j = 2) and 0 otherwise, ${\mathrm{T}2}_{\mathrm{ij}}$ is 1 if outcome is measured for participant i at 12 months (j = 3) and 0 otherwise and ${\mathrm{Z}}_{\mathrm{i}}$ is a vector of baseline adjustment covariates.

#### Secondary outcomes

We will use linear regression for continuous outcomes and Poisson regression for binary and count outcomes. We will use GEE [103, 105] to estimate regression models for longitudinal data using an independence working correlation matrix. We will calculate all standard errors using the robust sandwich estimator [103, 105] to account for within-person and within-health coach correlation or any mis-specified variance structures. We will include an interaction between intervention arms and time indicators, and the primary timepoint for all secondary analyses will be 3 months following randomization and will include as covariates baseline levels of pain severity and all stratification variables.

#### Economic evaluation

A full economic evaluation of the CBT-CP-based interventions, compared to usual care, will be conducted, using the framework of cost-effectiveness, including the costs of implementation and maintenance, following best practice in economic evaluation [106, 107]. This analysis will be conducted for the 3 Kaiser Permanente clinical sites where the capture of all healthcare utilization is available through administrative data. Information on resources used to implement the intervention will come from the trial data collection instruments and from medical office staff, provider interviews, and study staff. All relevant resources used in the intervention delivery (e.g., training, counseling, fidelity assurance) will be included. Electronic health records data will be used to identify and classify healthcare encounters and prescription medications. Using the framework of cost-effectiveness, the incremental cost per additional patient with a MCID in pain severity (30% reduction from baseline) will be estimated at 12 months, and the QALY gained—utilities will be estimated using the EQ-5D-5L [87].

##### Costs to be collected

Medical care utilization and intervention costs will be considered. Medical care utilization includes pharmacy, outpatient visits (including specialty care), and inpatient stays and will be costed using standard costing algorithms [74, 75] and Medicare fee schedules. In addition to total medical care costs, we will also undertake an analysis of pain-related care focused on utilization linked to pain conditions, identified by diagnostic and procedure codes, and pain-related medications. Intervention costs include program implementation (e.g., training, meetings, and supervision; patient identification, invitation, and screening) and delivery (e.g., online hosting, clinician calls). The analysis will take the perspective of the health plan (a principal decision maker for future implementation), so it will include all health system costs of intervention implementation and delivery in clinical settings.

##### Cost-effectiveness calculations

As done in prior economic evaluations of trials [108], the cost-effectiveness of the intervention will be estimated using net benefit regression methods [109, 110]. This technique uses a “net benefits” framework, comparing the incremental cost-effectiveness ratio to a range of potential values for a decision maker’s willingness-to-pay (WTP) for a unit of health gain. A cost-effectiveness acceptability curve (CEAC) is constructed that illustrates the intervention’s probability of being cost-effective at various levels of WTP for a unit of outcome (e.g., cost per QALY of $30,000 to $100,000). The regression framework allows ready evaluation of cost-effectiveness in subgroups (following the intervention’s findings). Net benefit regression uses as the dependent variable, net benefit: nb_i_ = λ·effect_i_ − cost_i_ (from person-level effect and cost data; λ = WTP level and is varied to construct the CEAC). Sensitivity analyses will be performed to assess the applicability of costs to other settings, the estimation of replication costs, and economies of scale [111].

##### Healthcare cost comparisons

A relative comparison of the healthcare costs between the randomized groups will be conducted. These comparisons will include overall, and pain-specific costs, which will be modeled separately. Healthcare costs will be modeled using general linear models with a gamma distribution and log link. Model specification will be a simplification of the model detailed above for the primary outcome and can be written as:

$${\mathrm{Y}}_{\mathrm{i}} = {\upbeta }_{0} +{\upbeta }_{1}\mathrm{Int}{1}_\mathrm{i }+ {\upbeta }_{2}\mathrm{Int}{2}_{\mathrm{i}} + {\upbeta }_{3}{\mathrm{BLC}}_{\mathrm{i}}+{\upbeta }_{\mathrm{z}}{\mathrm{Z}}_{\mathrm{i}}$$

where Y_i_ is follow-up healthcare costs for participant i (i = 1,…,n), Int1_i_ is 1 if the participant i is randomized to the first intervention group and 0 otherwise, Int2_i_ is 1 if participant i is randomized to the second intervention group and 0 otherwise, Z_i_ is a vector of the baseline adjustment covariates included in the primary outcomes model, and BLC_i_ is the additional baseline adjustment of baseline costs in the year prior to randomization.

### Interim analysis {21b}

Interim analyses will not be conducted as no potentially serious outcomes are expected.

### Methods for additional analyses (e.g., subgroup analyses) {20b}

Subgroup analyses will be conducted to determine the impact of the active interventions on specific populations and explore for potential heterogeneity of treatment effects by sex, age, race/ethnicity, rural/medically underserved residency, multiple pain conditions, mental health disorders, and negative social determinants of health. Analyses will follow the same general approach as for the primary outcome but will be focused on assessing heterogeneity of treatment effects (subgroups). We will assess heterogeneous treatment effects by each potential moderator separately. For each moderator, a main effect for the moderator and an interaction between the moderator, intervention, and follow-up time, to estimate time-specific intervention effects within each subgroup defined by the potential moderator. The primary comparison will be of the interaction terms associated with each intervention arm at the 3-month follow-up time. The longitudinal nature of data collection will allow us to qualitatively assess if the treatment effectiveness pattern is different over time in each of the intervention groups at the different levels of each of the moderators.

Mediation analyses will also be conducted to assess and quantify the effect of theory-based mediators (pain catastrophizing, pain self-efficacy, perceived support). Mediators represent a causal pathway between the intervention and outcome. Mediation occurs when the intervention influences a variable (the mediator) that in turn subsequently influences the outcome variable. Controlling for a mediator variable causes the strength of relationship between intervention and outcome to be meaningfully reduced. Consistent with recommendations, we will conduct mediation analyses only for interventions that have significant impacts on the outcomes under consideration at 6 months. Primary mediation analyses will assess the effect of the potential mediators on the primary outcome at 6 months, MCID in pain severity, while explanatory secondary analyses will investigate mediator impacts on secondary outcomes at 6 months. We will conduct mediation analyses using the framework recommended by Baron and Kenney [112] but using more recent statistical methods developed to better quantify and decompose different aspects of the mediation effect [113]. We will run a separate set of mediator analyses for each intervention compared to usual care. We will illustrate our approach for the binary outcome, achieving MCID in pain severity at 6 months.

### Methods in analysis to handle protocol non-adherence and any statistical methods to handle missing data {20c}

As the primary analysis, all effectiveness outcome measures are analyzed under intention-to-treat (ITT). A per-protocol sample may be constructed if there is crossover among greater than 10% of participants randomized to usual care (i.e., > 10% of usual care group receives CBT-CP) in which treatment-as-received is analyzed.

Our primary approach to handle missing data is through baseline adjustment which assumes that missing data to be missing at random (MAR) given the adjustment of the baseline covariates. If we observe > 15% missing primary outcome at 3 months, or differential missingness by group (> 10% difference between arms), we will conduct missing data sensitivity analyses. We will address missing data in 2 ways. Our first approach will apply a pattern mixture imputation missing data approach that relaxes the MAR assumption conditioning on the patterns of missing data over time [114]. The second approach will be used as a sensitivity analysis assuming a worst-case, best-case approach. Specifically, for those with missing outcome data in the intervention groups, we will assume they did not achieve the MCID in pain severity at each missing time point (worst-case). For those randomized to the usual care, we will assume all achieved the MCID in pain severity at each missing time point (best-case). This sensitivity analysis will provide the extreme case in how small the intervention effect could be relative to usual care due to missing outcome data.

### Plans to give access to the full protocol, participant-level data, and statistical code {31c}

The full protocol will be accessible on ClinicalTrials.gov at the time the study results are posted. All data collection forms are available from the corresponding author on request. A database for underlying primary data for publications will be made publicly available on the NIH HEAL Initiative central data repository. The database will be de-identified in accordance with the definitions provided in the HIPAA and will be accompanied by a data dictionary that provides a concise definition of every data element included in the database. The policies for release of and access to this database are in accordance with the HEAL Data Sharing policy as determined by the NIH. The statistical code for primary study analyses will be available from HEAL Pain Effectiveness Research Network Trial Innovation Center (TIC) at Johns Hopkins University which serves as the Biostatistics Core for the study.

## Oversight and monitoring

### Composition of the coordinating center and trial steering committee {5d}

Study monitoring is conducted by Trial Innovation Centers (TICs) of the NCATS Trial Innovation Network. Specifically, the Duke University TIC serves as Clinical Coordinating Center (CCC) and the University of Utah TIC serves as the Data Coordinating Center (DCC). Clinical and remote site monitoring is conducted to ensure that the rights of human subjects are protected, that the study is implemented in accordance with the protocol and/or other operating procedures, and that the quality and integrity of study data and data collection methods are maintained.

### Composition of the data monitoring committee, its role and reporting structure {21a}

Study oversight is under the direction of a Data and Safety Monitoring Board (DSMB) comprised of experts from chronic pain treatment, behavioral interventions, and statistics. Specifically, the DSMB members include David A. Williams, PhD (Chair), Steven Dobscha, MD, and Keith Goldfeld, DrPH. The DSMB met before study enrollment began to approve the study protocol prior to implementation and meets biannually until the study ends to assess safety and effectiveness data, monitor accrual of study participants, and assess study progress and data integrity for the study. If safety concerns arise, ad hoc meetings and more frequent standing meetings of the DSMB may be held. The DSMB operates under the rules of a charter that was approved at the initial meeting. The Johns Hopkins University TIC serves as the DSMB Unit for the RESOLVE study, supporting the DSMB members, as an independent entity, in the organization and implementation of successful DSMB meetings and is composed of a DSMB unit director, safety officer, DSMB coordinator, biostatistician, and an independent analyst. The DSMB will provide recommendations to the funding agency, the National Institute on Aging (NIA), regarding proceeding with the study as planned, proceeding with modifications, or terminating the study, as noted in the DSMB Charter.

### Adverse event reporting and harms {22}

Adverse events (AEs) are identified by participant self-report, and select serious adverse events (SAEs), inpatient hospitalizations and deaths, are systematically assessed based on electronic health records and healthcare system administrative data. Every 6 months, each clinical site queries active participants’ electronic health records data (in alignment with the DSMB meeting schedule) to identify any hospitalizations or deaths throughout the interval of active participant enrollment in the trial. An independent physician within each healthcare system conducts a chart review for each death to assess its potential relatedness to study procedures and interventions. The summary of SAEs and findings of these reviews are submitted to the DCC for reporting to the DSMB.

### Frequency and plans for auditing trial conduct {23}

Monitoring is performed by the Duke CCC of the NIH HEAL Pain ERN in collaboration with the Utah DCC. Data are reviewed twice a month throughout the active study period for completeness; timeliness with completing data entry, per responsibilities of the site; ensuring participant visits occur within the appropriate time frame for scheduled study activities; and logical data (i.e., no “unbelievable” values).

A remote “for cause” monitoring visit may be triggered if there are serious issues of concern, which may include serious or persistent non-compliance with the protocol, study requirements, and/or applicable regulations and guidelines.

### Plans for communicating important protocol amendments to relevant parties (e.g., trial participants, ethical committees) {25}

Approval for any study protocol amendments will be obtained from the Vanderbilt University Medical Center Institutional Review Board (IRB) which serves as the single IRB for the study. If warranted by the amendment, participant consent materials and information sheets will be updated accordingly and any changes to the published protocol will be reported in full in any future publications.

### Dissemination plans {31a}

The results from this clinical trial will be fully disclosed by means of publications in international peer-reviewed journals and by oral/poster presentations at national and international scientific meetings. Both positive and negative findings will be disclosed.

## Discussion

The RESOLVE comparative effectiveness clinical trial addresses one of the most pervasive and costly public health issues in the U.S.: how to treat high-impact chronic pain in a safe and both clinically and cost-effective manner that can be disseminated in areas of the country lacking local healthcare providers who have the expertise and training to deliver CBT-CP. Further, the scale of the study and inclusion of two active treatment arms allows us to consider the comparability of two widely accepted, remote forms of CBT-CP-based treatment delivery, including in what circumstances a less resource-intensive online program may be sufficient, and when those with more complex clinical presentations or other socio-demographic characteristics may achieve better outcomes with a telehealth version of CBT-CP. In addition, the range of secondary outcomes includes those highly valued by patients beyond pain-specific domains—physical and social functioning as well as their global impression of change achieved through treatment [115]. The planned cost-effectiveness analyses and robust qualitative analyses will further help us identify the circumstances under which one or the other may be preferrable for adoption.

Although this study was proposed and funded prior to the emergence of COVID-19, the pandemic induced shift towards online and telehealth-based clinical services for conditions like chronic pain renders the core RESOLVE study questions particularly timely and salient [116, 117]. During 2020, remote intervention delivery in the mental health services market increased by 4300% [118]. Further, 20% of all Medicare and Medicaid services are expected to be provided through telehealth post-pandemic. Significantly, many of the previous barriers to remote delivery of nonpharmacologic treatment for chronic pain like CBT-CP (e.g., insurance coverage and reimbursement, practicing across state lines) were lowered during the pandemic and these changes are likely to be sustained [44, 119]. Nonetheless, barriers remain, including variable access to high-speed internet, particularly in rural areas of the country; lack of patient and clinician familiarity and comfort with remote treatment; and sometimes cumbersome security and privacy safeguards for the technical platforms supporting such care [45, 116]. While the RESOLVE study is not designed to address such barriers, our qualitative component should help us illuminate issues that should be addressed to optimize feasibility and promote widespread adoption of remote CBT-CP interventions. Collectively, the rapid adoption of remote modalities underscores the importance of better understanding the effectiveness, acceptability, and limitations of interventions such as those under consideration in RESOLVE.

In summary, this large-scale comparative effectiveness trial provides a unique opportunity to rigorously evaluate and compare the clinical and cost-effectiveness of two relatively low-cost, scalable modalities for providing evidence-based CBT-CP treatments. The RESOLVE study addresses the urgent need to make CBT-CP widely available, particularly for patients residing in rural and medically underserved areas who have been disproportionally affected by the opioid crisis. Centralizing delivery of the CBT-CP-based programs via telephone/videoconferencing and online interventions, if successful, could encourage timely scalability and widespread dissemination into frontline clinical care and healthcare organizations nationwide.

## Trial status

This is version 5.0 of the RESOLVE trial protocol, dated November 10, 2022. Recruitment for the trial began on January 12, 2021, under protocol version 2.0, dated November 24, 2020. Recruitment is expected to be completed in the first quarter of 2023.

## Data Availability

The study protocol has been reported in accordance with the Standard Protocol Items: Recommendations for Clinical Interventional Trials (SPIRIT) guidelines. The data collection forms are available from the corresponding author on request. A database for underlying primary data for publications will be made publicly available on the NIH HEAL Initiative central data repository. The database will be de-identified in accordance with the definitions provided in the Health Insurance Portability and Accountability Act (HIPAA) and will be accompanied by a data dictionary that provides a concise definition of every data element included in the database. The policies for release of and access to this database are in accordance with the HEAL Data Sharing policy as determined by the NIH.

## Abbreviations

AEs: Adverse events
BPI-SF: Brief Pain Inventory-Short Form
CBT: Cognitive behavioral therapy
CBT-CP: Cognitive behavioral therapy for chronic pain
COVID-19: Coronavirus disease of 2019
CEAC: Cost-effectiveness acceptability curve
HIPAA: Health Insurance Portability and Accountability Act
HEAL: Helping to End Addiction Long-term®
IRB: Institutional Review Board
ITT: Intention-to-treat
KP: Kaiser Permanente
KPGA: Kaiser Permanente Georgia
KPNW: Kaiser Permanente Northwest
KPWA: Kaiser Permanente Washington
MCID: Minimal clinically important difference
MAR: Missing at random
NCATS: National Center for Advancing Translational Science
NIH: National Institutes of Health
QALYs: Quality-adjusted life years
SAEs: Serious adverse events
WTP: Willingness-to-pay
